# Supplementary material for: Mapping of Determinants of Urinary Sex Steroid Metabolites During Late Pregnancy: Results from Two Spanish Cohorts
Source: Int J Mol Sci. 2025 Nov 29;26(23):11598. doi: 10.3390/ijms262311598 (PMC12692236; doi:10.3390/ijms262311598)
Supplement: Supplementary file 1 [file ijms-26-11598-s001.zip › Supplementary-figures_07112025.pdf]

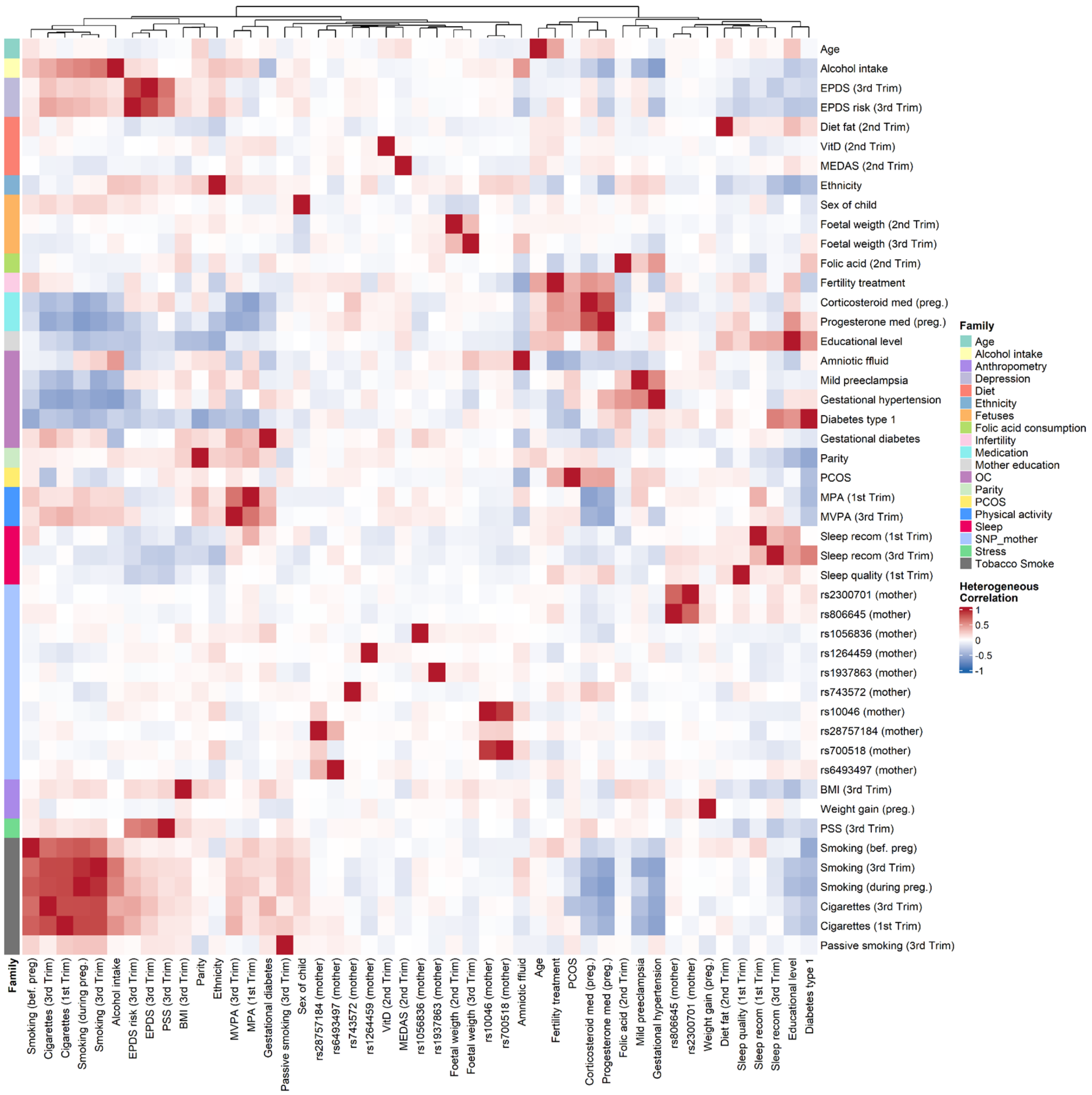

Figure S1. Heatmap correlation between determinants from BiSC cohort (n=721)

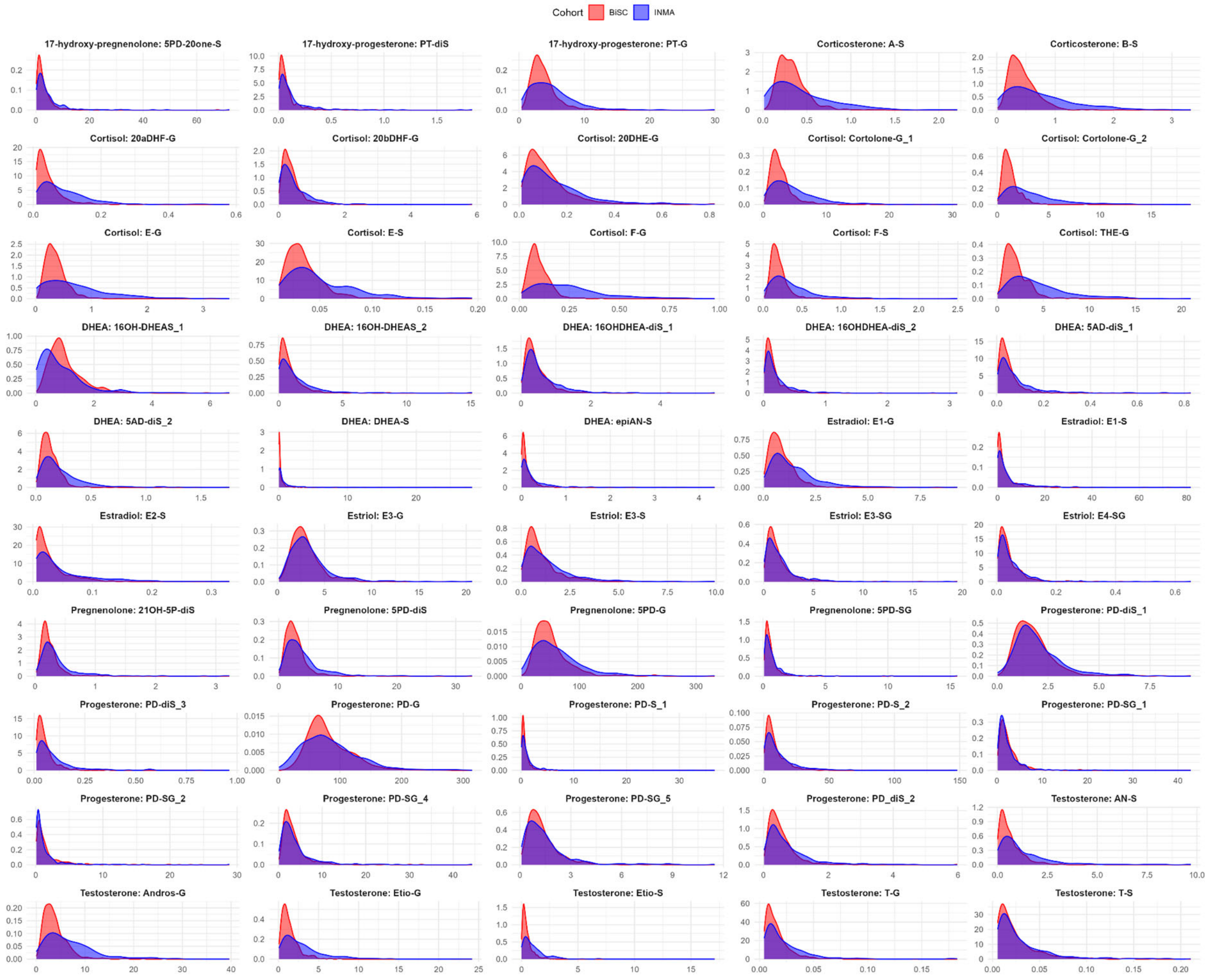

Figure S2. Raw SH concentration (50 metabolites **µ**mol/L) from BiSC cohort (n=721) and INMA cohort (n=500).

DHEA-S, Dehydroepiandrosterone (DHEA)-sulfate; 5AD-diS_1, 5-androsten-3b17b-diol-diSulfate; 5AD-diS_2, 5-androsten-3a17b-diol-diSulfate; 16OHDHEA-diS_1, 16b-hydroxy-DHEA-diSulfate; 16OHDHEA-diS_2, 16a-hydroxy-DHEA-diSulfate; epiAN-S, epiandrosterone-Sulfate; 16OH-DHEAS_1, 16b-hydroxy-DHEA-Sulfate; 16OH-DHEAS_2, 16a-hydroxy-DHEA-Sulfate; T-G, Testosterone-Glucuronide; T-S, Testosterone-Sulfate; AN-S, Androsterone-Sulfate; Etio-S, Etiocholanolone-Sulfate; Andros-G, Androsterone-Glucuronide; Etio-G, Etiocholanolone-Glucuronide; B-S, Corticosterone-Sulfate; A-S, 11-dehydrocorticosterone-Sulfate; F-S, Cortisol-Sulfate; E-S, Cortisone-Sulfate; E-G, Cortisone-Glucuronide; F-G, Cortisol-Glucuronide; 20DHE-G, 20dihydrocortisone-Glucuronide; 20aDHF-G, 20a-dihydrocortisol-Glucuronide; 20bDHF-G, 20b-dihydrocortisol-Glucuronide; THE-G, Tetrahydrocortisone-Glucuronide; Cortolone-G_1, 20a-Cortolone-Glucuronide; Cortolone-G_2 20b-Cortolone-Glucuronide; E2-S, Estradiol-Sulfate; E1-G, Estrone-Glucuronide; E1-S Estrone-Sulfate; E3-G, Estriol-Glucuronide; E3-SG, Estriol-Sulfoglucoconjugated; E3-S, Estriol-Sulfate; E4-SG, Estetrol-Sulfoglucoconjugated; 5PD-20one-S, 17-hydroxy-5-pregnenolone-3-sulfate; PT-diS, Pregnantriol-diSulfate; PT-G, Pregnantriol-Glucuronide; 5PD-diS, 5-Pregnendiol-DiSulfate; 21OH-5P-diS, 21-Hydroxypregnenolone-DiSulfate; 5PD-SG, 5-Pregnendiol-Sulfoglucoconjugated; 5PD-G, 5-Pregnendiol-Glucuronide; PD-diS_1, 5a-Pregnan-3b,20a-diol-DiSulfate; PD_diS_2, 5a-Pregnan-3a,20a-diol-DiSulfate; PD-diS_3, 5b-Pregnan-3a,20a-diol-DiSulfate; PD-SG_1, 5a-Pregnandiol-3b-sulfate-20a-Glucuronide; PD-SG_2, Pregnandiol-sulfoglucoconjugate; PD-SG_4, Pregnandiol-sulfoglucoconjugate; PD-SG_5, Pregnandiol-sulfoglucoconjugate; PD-S_1, 5a-Pregnan-3b20a-diol-20-Sulfate; PD-S_2, 5b-Pregnan-3a20a-diol-20-Sulfate; PD-G, Pregnandiol-Glucuronide.

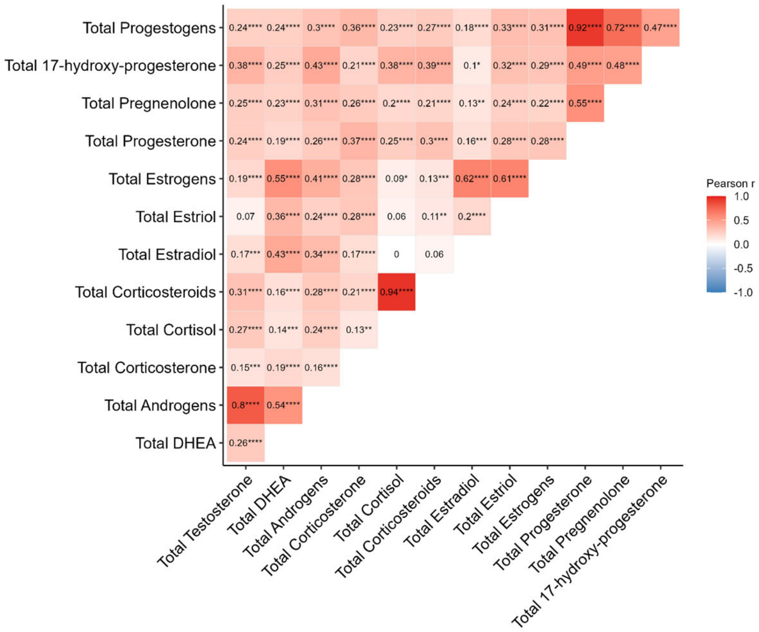

Figure S3. Heatmap correlation between SH metabolome from BiSC cohort (n=721)
**** P<0.0001, *** P < 0.001, ** P < 0.01, * P< 0.05

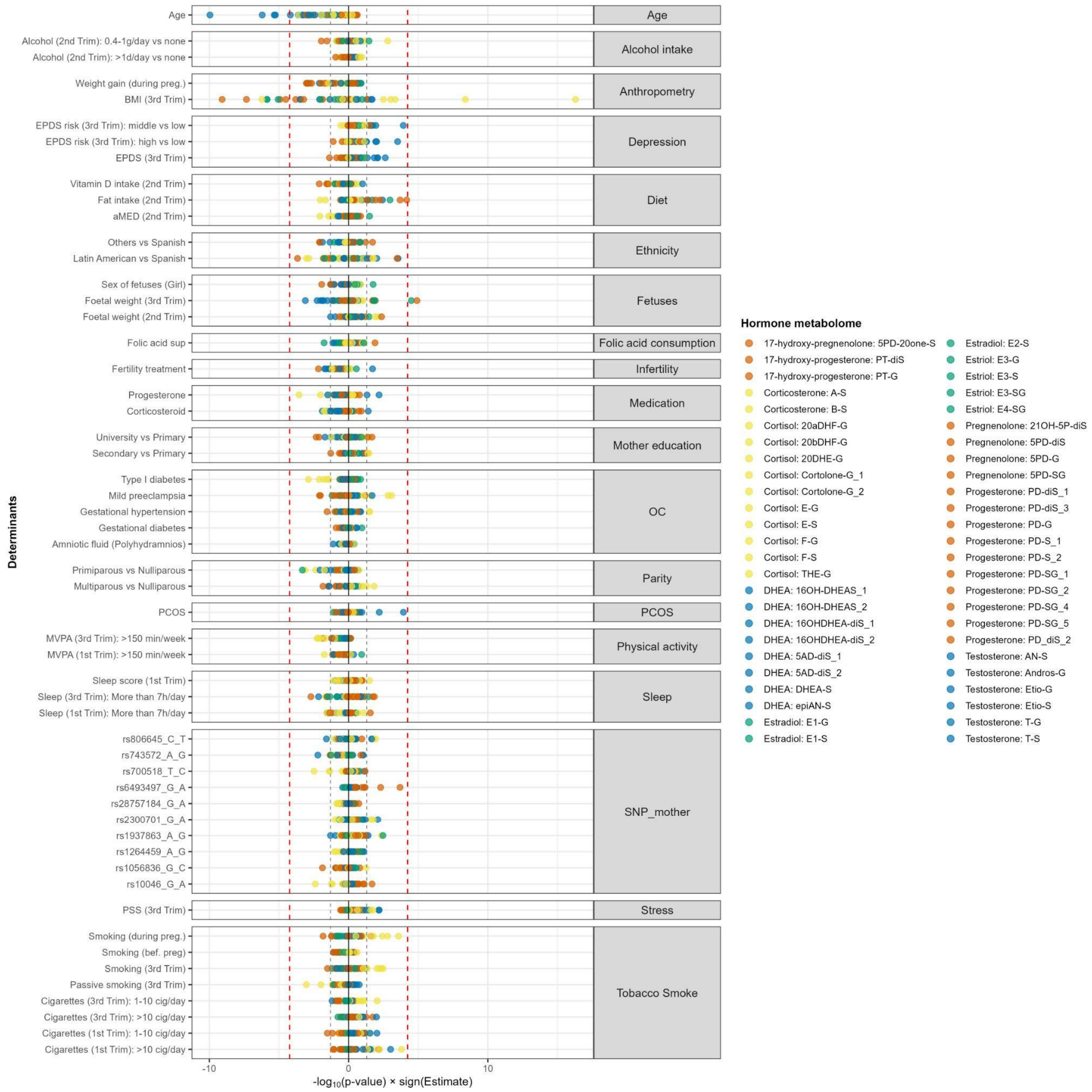

Figure S4. Miami-plot from ExWAS Analysis of Main Determinants and the Steroid Metabolome in the BISC Cohort (n=721)

Results of an ExWAS between key determinants and the steroid metabolome. The main determinants include physiological factors (mother and fetus), sociodemographic variables, genetics (polymorphism of steroid hormones enzymes), medical history, stress, depression, and lifestyle factors (alcohol intake, smoking, dietary intake, physical activity, and sleep pattern). IQR normalization of determinants was applied. The steroid metabolome was log2 transformed. All associations were adjusted for potential confounding variables, including hospital in the 3rd trimester or at birth, COVID-19 exposure period, and season of birth. *P* outside the red threshold line is significant after correction for the effective number of tests (ENT). ENT for SH metabolites was $P < 0.00006$.

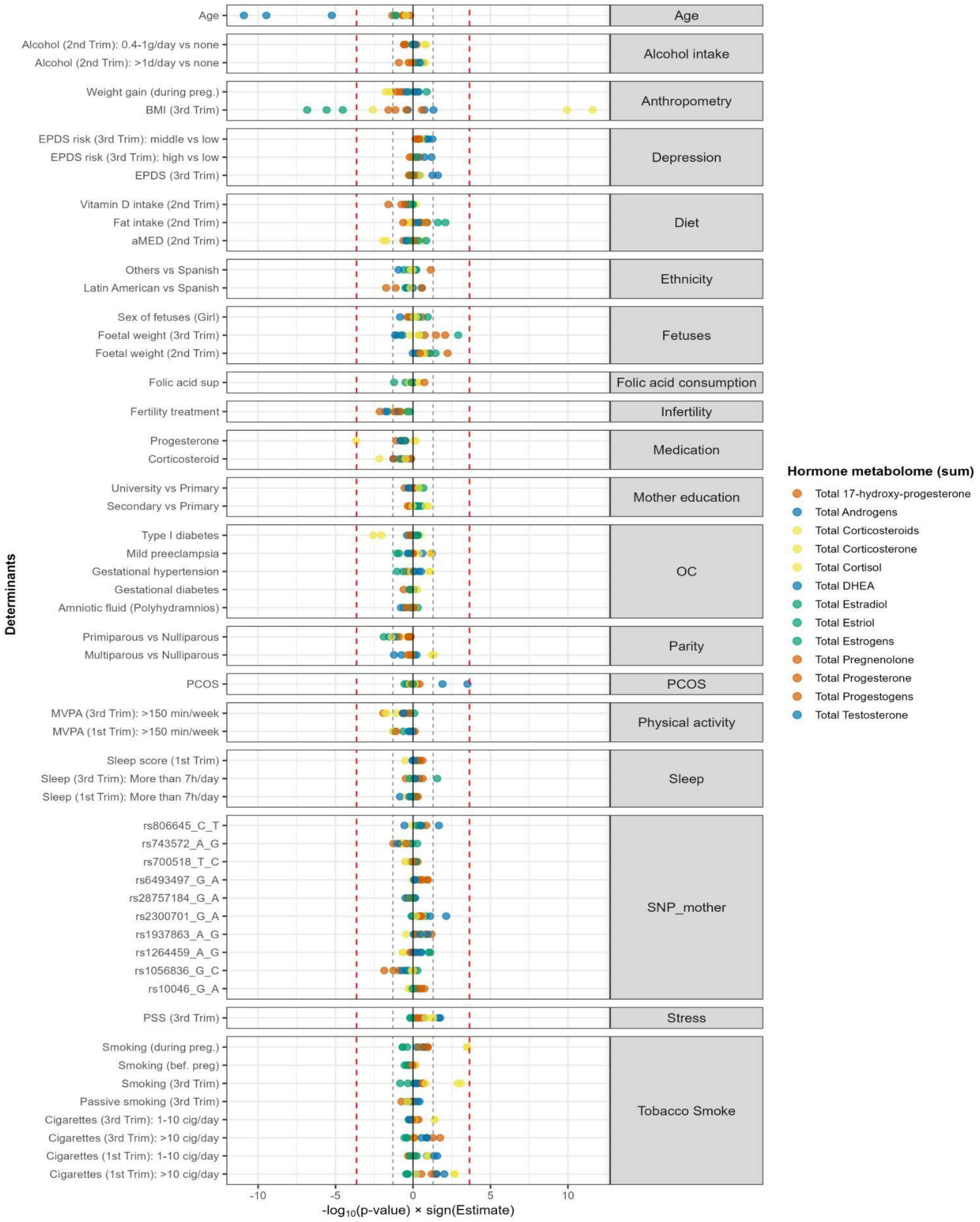

Figure S5. Miami-plot from ExWAS Analysis of Main Determinants and the Sum of Steroid Metabolome in the BISC Cohort (n=721)

Results of an ExWAS between key determinants and the steroid metabolome. The main determinants include physiological factors (mother and fetus), sociodemographic variables, genetics (polymorphism of steroid hormones enzymes), medical history, stress, depression, and lifestyle factors (alcohol intake, smoking, dietary intake, physical activity, and sleep pattern). IQR normalization of determinants was applied. The steroid metabolome was log2 transformed. All associations were adjusted for potential confounding variables, including hospital in the 3rd trimester or at birth, COVID-19 exposure period, and season of birth. *P* outside the red threshold line is significant after correction for the effective number of tests (ENT). ENT for sum was *P* <0.0002.

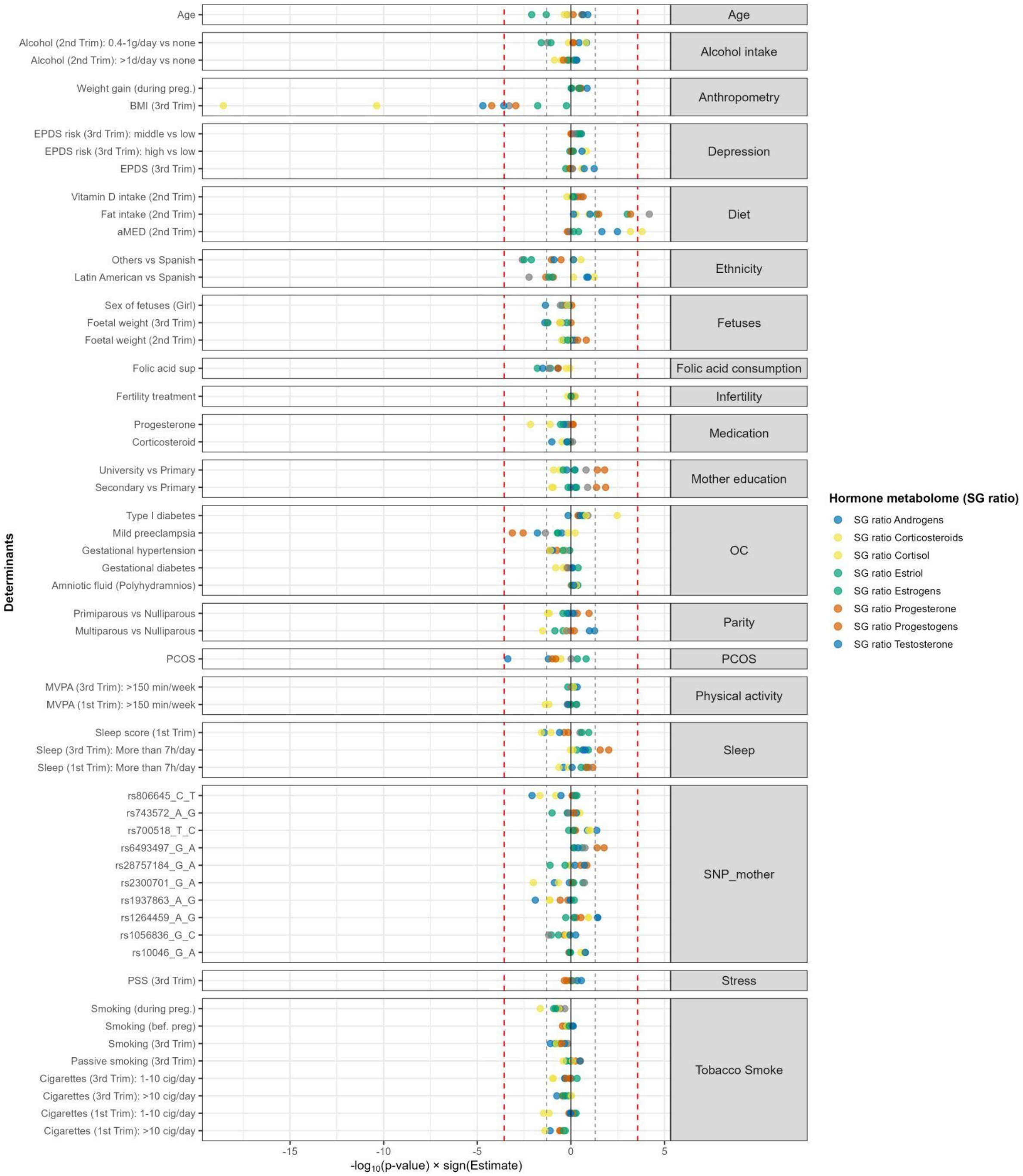

Figure S6. Miami-plot from ExWAS Analysis of Main Determinants and the Sulfate-glucuronide Steroid Metabolome ratios in the BISC Cohort (n=721)

Results of an ExWAS between key determinants and the steroid metabolome. The main determinants include physiological factors (mother and fetus), sociodemographic variables, genetics (polymorphism of steroid hormones enzymes), medical history, stress, depression, and lifestyle factors (alcohol intake, smoking, dietary intake, physical activity, and sleep pattern). IQR normalization of determinants was applied. The steroid metabolome was log2 transformed. All associations were adjusted for potential confounding variables, including hospital in the 3rd trimester or at birth, COVID-19 exposure period, and season of birth. *P* outside the red threshold line is significant after correction for the effective number of tests (ENT). ENT for SG ratio was *P*< 0.0003.

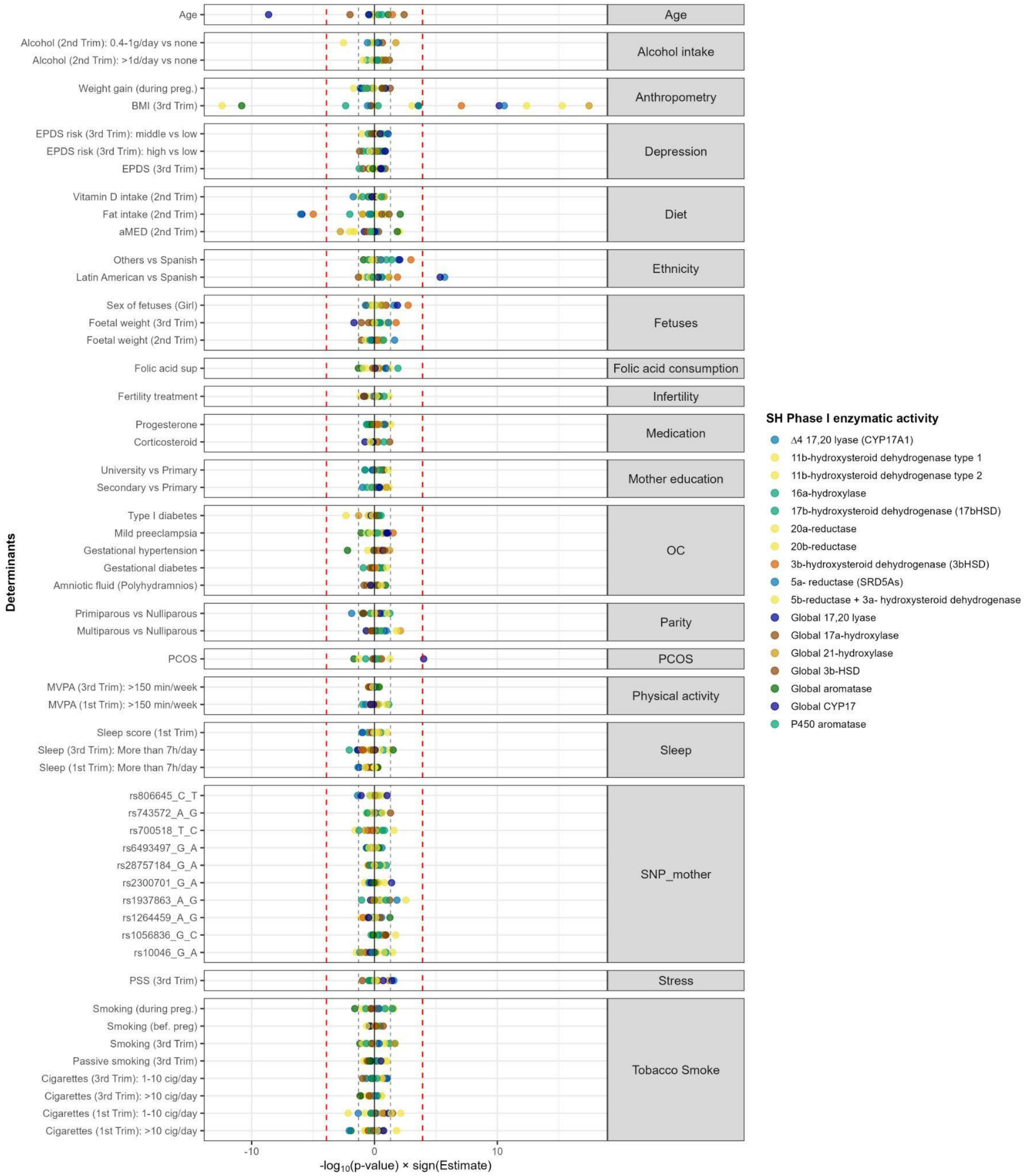

Figure S7. Miami-plot from ExWAS Analysis of Main Determinants and the Phase I Steroid Metabolome enzymatic activity in the BISC Cohort (n=721)

Results of an ExWAS between key determinants and the steroid metabolome. The main determinants include physiological factors (mother and fetus), sociodemographic variables, genetics (polymorphism of steroid hormones enzymes), medical history, stress, depression, and lifestyle factors (alcohol intake, smoking, dietary intake, physical activity, and sleep pattern). IQR normalization of determinants was applied. The steroid metabolome was log2 transformed. All associations were adjusted for potential confounding variables, including hospital in the 3rd trimester or at birth, COVID-19 exposure period, and season of birth. *P* outside the red threshold line is significant after correction for the effective number of tests (ENT). ENT for phase I enzymatic activity was *P*< 0.0001.

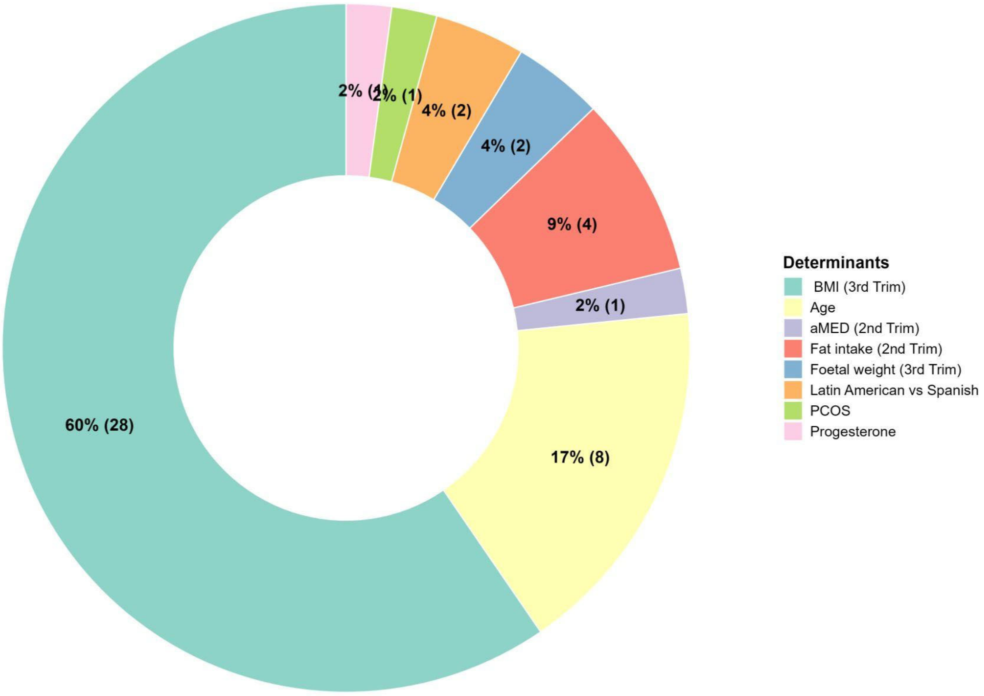

Figure S8: Proportion of significant ExWAS associations after multi testing correction in the BISC Cohort (n=721).

All associations were adjusted for potential confounding variables, including hospital in the 3rd trimester or at birth, COVID-19 exposure period, and season of birth. P significant after correction for the effective number of tests for SH metabolites was $P < 0.00006$, sum was $P < 0.0002$, SG ratio was $P < 0.0003$, phase I enzymatic activity was $P < 0.0001$.

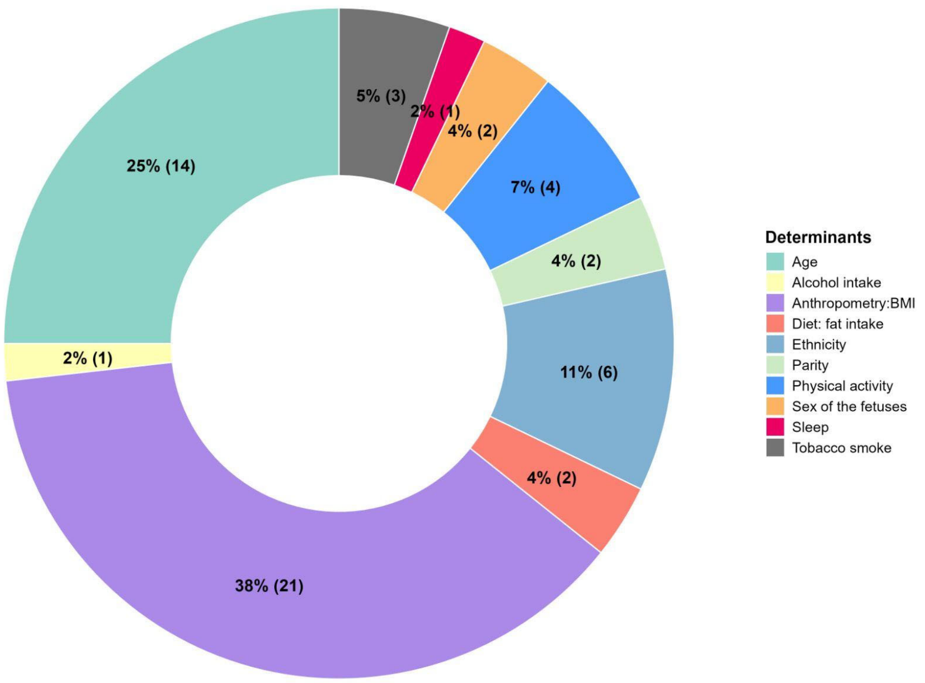

Figure S9: Proportion of concordant determinant-SH molecular features pairs significantly associated in the BISC (n=721) and INMA-Sabadell (N=500) Cohorts.
